# Expression of amphiregulin predicts poor outcome in patients with pancreatic ductal adenocarcinoma

**DOI:** 10.1186/s13000-016-0512-4

**Published:** 2016-07-08

**Authors:** Li Wang, Huanwen Wu, Lili Wang, Junliang Lu, Huanli Duan, Xuguang Liu, Zhiyong Liang

**Affiliations:** Molecular Pathology Research Center, Department of Pathology, Peking Union Medical College Hospital, Chinese Academy of Medical Sciences and Peking Union Medical College, Beijing, 100730 People’s Republic of China

**Keywords:** EGFR, EGFRvIII, AREG, Pancreatic ductal adenocarcinoma

## Abstract

**Background:**

The validation of novel diagnostic, prognostic and predictive biomarkers in cancer is crucial for optimizing the choice and efficacy of personalized therapies. The aim of this study was to determine the epidermal growth factor receptor (EGFR), epidermal growth factor receptor variant III (EGFRvIII) and amphiregulin (AREG) protein expression levels and to evaluate the prognostic significance of EGFR, EGFRvIII and AREG in pancreatic ductal adenocarcinoma (PDAC).

**Methods:**

The EGFR, EGFRvIII and AREG protein levels in PDAC (*n* = 92) were examined by using immunohistochemistry. The associations between EGFRvIII expression, AREG expression, AREG/EGFR co-expression and clinicopathological factors were assessed, the correlation between AREG and EGFR expression was analyzed and the survival analyses were performed.

**Results:**

Among the lesions of PDAC, 12 (13 %) stained positive for EGFRvIII, 49 (53.3 %) stained positive for AREG and 22(23.9 %) stained double positive for AREG/EGFR. The relationships between each protein expression level and the clinicopathologic factors were examined, only AREG/EGFR co-expression was significantly related to tumor differentiation (*P* = 0.032). The correlation between AREG and EGFR expression was statistically insignificant (*P* = 0.709). Univariate survival analysis proved that high tumor-node-metastasis (TNM) stage, poor tumor differentiation and AREG expression were significant poor prognostic factors for disease-free survival (DFS) and overall survival (OS). By multivariate survival analysis, tumor differentiation was an independent poor prognostic factor for DFS (HR = 1.785, *P* < 0.05), whereas high TNM stage (HR = 2.25, *P* < 0.05), poor tumor differentiation (HR = 2.125, *P* < 0.01), positive resection margins (HR = 1.84, *P* < 0.05), and AREG expression (HR = 1.822, *P* < 0.05) were all independent poor prognostic factors for OS.

**Conclusions:**

In conclusion, our data indicate that AREG expression is an important prognostic biomarker in PDAC .

**Electronic supplementary material:**

The online version of this article (doi:10.1186/s13000-016-0512-4) contains supplementary material, which is available to authorized users.

## Background

Pancreatic ductal adenocarcinoma (PDAC) is the fourth leading cause of cancer-related deaths in the United States and the sixth leading cause of cancer-related deaths for males in China with a 5-year survival rate of less than 7 % [1, 2]. The majority of PDAC patients are diagnosed at an advanced stage and thus are not candidates for treatment with curative intent. Because PDAC patients usually show partial responses to traditional cytotoxic chemotherapy, specific molecule inhibition represents an attractive target for cancer therapy. The epidermal growth factor receptor (EGFR) has been increasingly recognized as a molecular target in cancer therapy. The combination of gemcitabine and erlotinib was the first combination therapy to demonstrate survival benefits in advanced or metastatic pancreatic cancer in a phase III, double-blind, placebo-controlled study [3]. As a result, gemcitabine-erlotinib combination therapy was approved by the Food and Drug Administration (FDA) for the first-line treatment of patients with locally advanced non-resectable or metastatic pancreatic cancer.

Dysregulated EGFR signaling (such as cell-surface overexpression, autocrine activation and EGFR gene mutation) contributes to the formation of several epithelial malignancies in humans [4]. There is increasing recognition that epidermal growth factor receptor variant III (EGFRvIII), the most common form of mutant EGFR, is an important target for cancer therapy. EGFRvIII comprises an in-frame deletion of 267 amino acids from the extracellular domain of EGFR. Although it is unable to bind ligand, EGFRvIII shows a low-level constitutive kinase activity and impaired endocytosis and degradation [5]. EGFRvIII is not detected in normal tissues, while it is overexpressed in several cancer types, particularly glioblastoma multiforme (GBM) [6]. However, whether EGFRvIII is expressed in pancreatic cancer remains unclear.

Amphiregulin (AREG) is a member of ligand family of EGFR. After bind to the extracellular ligand-binding domain of EGFR, AREG activates intracellular signaling cascades governing cell survival, proliferation, and motility [7]. Accordingly, several studies have focused on the disruption of AREG-mediated oncogenic pathways. AREG is upregulated in various neoplasms including colon, lung, liver, breast, prostate, and pancreatic cancer [7]. Functional studies show that AREG is involved in most of the hallmarks of cancer [8–11]. It has also been reported that AREG expression is a promising predictive marker for liver metastasis in primary colorectal cancer [9]. Tinhofer et al. demonstrated that patients with squamous cell carcinoma of the head and neck (SCCHN) who showed high AREG expression were less likely to benefit from combination treatment with cetuximab and docetaxel [10]. However, reports regarding AREG expression in pancreatic cancer by Park showed that decreased expression of AREG was a typical characteristic of the tumor biology [11].

Therefore, the aims of our study were to investigate the expression of EGFRvIII, AREG and AREG/EGFR co-expression in resected PDAC tissues and to explore the clinicopathological and prognostic significance of their expression in PDAC.

## Methods

### Human tissues

Patients who had preoperative chemotherapy (CT) or radiotherapy (RT), macroscopic incomplete resection (R2), or inadequate follow-up data and a survival time of less than 30 days from the time of surgery were excluded in our study. The study population comprised 92 patients who underwent the resection for PDAC at Peking Union Medical College Hospital during the period between January 2009 and December 2014. The study protocol was approved by the Institutional Ethics Committee at Peking Union Medical College Hospital. Written informed consent was obtained from all patients at the time of their treatment for use of material in future research.

### Clinicopathologic data

The medical records of enrolled patients were reviewed from the pathologists’ electronic medical records system at Peking Union Medical College Hospital. They included the following data: age, sex, date of surgery, tumor location, tumor size, pathologic stage (tumor-node-metastasis, TNM stage), tumor differentiation, patterns and the site of recurrence, patterns of resection margins. Disease free survival (DFS) was determined from the time from surgery until local or metastatic PDAC tumor recurrence. Overall survival (OS) was defined as the time of surgery to death. The follow-up period after the initial operation for primary lesions was between 1 to 5 years.

### Immunohistochemistry

For the immunohistochemical study, 92 formalin-fixed, paraffin-embedded tumor specimens were collected. Conventional 4-μm sections from the tissue blocks were used. Immunohistochemistry was produced as previously described [12]. The slides were incubated with a monoclonal mouse anti-EGFR antibody (1:200 dilution; Santa Cruz), a monoclonal mouse anti-EGFRvIII antibody (1:200 dilution; Biorbyt), or a polyclonal goat anti-AREG antibody (1:50 dilution; R&D).

### Evaluation of immunostaining

For the membranous and/or cytoplasmic expression levels of EGFR and EGFRvIII, immunoreactivity was defined in the same manner as previously described [13]. The cytoplasmic expression levels of AREG was socred by applying a semi-quantitative immunoreactive score (IRS) system [14]. Briefly, immunostatining intensity was scored as: 0 = no staining, 1 = weak staining, 2 = moderate staining and 3 = strong staining. The extent of stained cells was stratified into three groups based on the percentage of positive cells: 0 = 0 %, 1 = 1–33 %, 2 = 33–66 %; and 3= > 66 %. IRS scores were obtained by multiplying the staining intensity by the number of group which ranging from 0 to 9. The final IRS scores >3 were considered AREG positive. The slides were independently evaluated by two of the authors (LW and HWW) to assess the protein expression levels.

### Statistical analysis

The categorical variables were compared using the *χ*^2^ test. The correlation between AREG and EGFR expression was examined by Pearson’s test. Kaplan-Meier survival curves were used to estimate the disease-free survival (DFS) and overall survival (OS) of the patients which was determined using the log-rank test. Multivariate analysis was performed by Cox proportional hazard regression mode. Two-sided *P* values < 0.05 were considered significant. All statistical procedures were performed with SPSS software for Windows, version 17.0 (SPSS Inc., Chicago, IL, USA).

## Results

### Patient characteristics and Immunohistochemical analysis

Among the 92 PDAC patients, there were 50 men and 42 women with a median age of 61 years (range: 34-80 years). The patient characteristics were described in Table 1.

**Table 1:** Summary of baseline patient characteristics (*n* = 92)

Characteristic	Variable	Value
Age range (years)		34–80
	Median	61
	Mean	60
Sex	Male vs. Female	50:42 (54.3 vs. 45.7 %)
Tumor location	Head vs. Body/tail	52:40 (56.5 vs. 43.5 %)
Tumor size	T1-2 vs. T3-4	23:69 (25 vs. 75 %)
TNM stage	I-II vs. III-IV	80:12 (87 vs. 13 %)
Tumor differentiation	Well/moderate vs. Poor	63:29 (68.5 vs. 31.5 %)
Lymph node metastasis	Yes vs. No	51:41 (55.4 vs. 44.6 %)
Resection margins	Positive vs. Negative	18:74 (20 vs. 80 %)

**Fig. 1:**
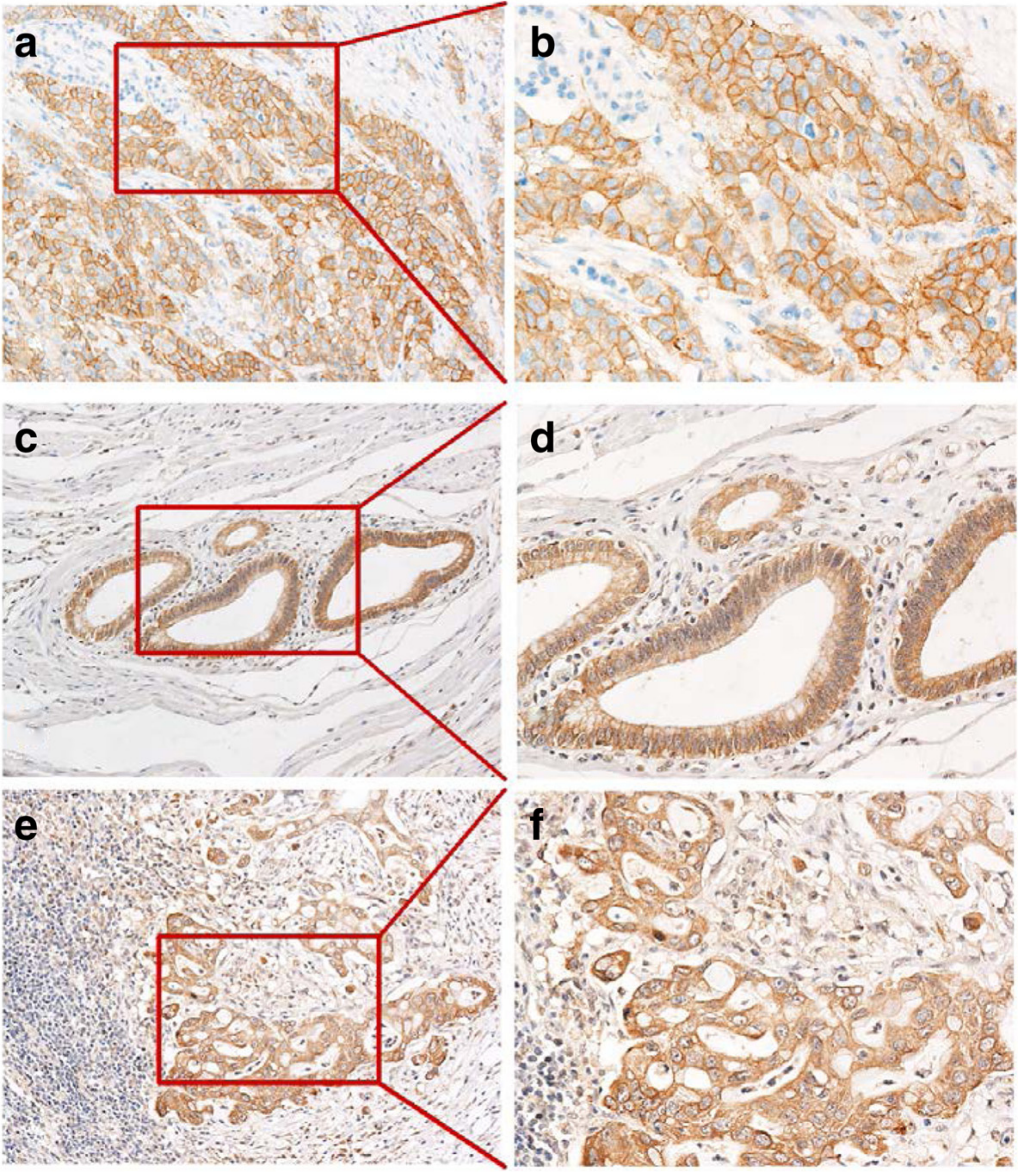
Detection of EGFR, EGFRvIII, AREG expression in PDAC. **a** representative tumor samples with EGFR expression, ×100. **b** EGFR expression, ×200. **c** EGFRvIII expression, ×100. **d** EGFRvIII expression, ×200. **e** AREG expression, ×100. **f** AREG expression, ×200

In total, EGFRvIII expression of PDACs was 12 (13 %), AREG expression of PDACs was 49 (53.3 %) and AREG/EGFR co-expression of PDACs was22 (23.9 %). EGFR and EGFRvIII was predominantly localized at the cellular membrane, AREG were mainly detected in the cytoplasm. Representative PDAC tissues with EGFR, EGFRvIII and AREG expression profiles are shown in Fig. 1. The acinar and ductal cells of the peritumoral areas showed negative or weak staining for EGFR and AREG, whereas these proteins were weakly or moderately expressed in normal pancreatic islet cells. Weak or moderate staining for AREG was also observed in a majority of the fibroblasts.

A significant association between AREG/EGFR co-expression and tumor differentiation was observed in our study (*P* = 0.032). However, there was no significant association between EGFRvIII expression or AREG expression alone and clinicopathological characteristics in PDAC, (Table 2).

**Table 2:** Correlation between EGFRvIII expression, AREG expression, AREG/EGFR co-expression and clinicopathologic factors in PDAC

Parameter	EGFRvIII		*P*	AREG		*P*	AREG/EGFR		*P*
	Negative	Positive		Negative	Positive		Negative^a^	Positive^b^	
Overall	80	12		43	49		70	22	
Age(years)			0.056			0.241			0.65
< 60	30	8		15	23		28	10	
≥ 60	50	4		28	26		42	12	
Gender			0.124			0.566			0.057
Male	39	3		22	28		34	16	
Female	41	9		21	21		35	6	
Tumor sites			0.625			0.256			0.09
Head	46	6		27	25		43	9	
Body/tail	34	6		16	24		27	13	
Tumor size			0.475			0.117			0.158
T1-2	21	2		14	9		20	3	
T3-4	59	10		29	40		50	19	
TNM stage			0.187			0.106			0.925
I-II	71	9		40	40		61	19	
III-IV	9	3		3	9		12	3	
Tumor differentiation			0.885			0.803			0.032
Well/morderate	55	8		30	33		52	11	
Poor	25	4		13	16		18	11	
Resection margins			0.067			0.457			0.851
Negative	62	12		36	38		56	18	
Positive	18	0		7	11		14	4	
Lymph node metastasis			0.828			0.725			0.923
No	36	5		20	21		31	10	
Yes	44	7		23	28		39	12	

^a^Negative: single positive or dual-negative for AREG and EGFR

^b^Positive: AREG/EGFR coexpression(double positive for AREG and EGFR)

### Correlation between AREG and EGFR expression

Among 92 PDAC patients, 22 were positive for AREG and EGFR expression, 21 were single-positive for EGFR, 27 were single-positive for AREG, and 22 were negative for AREG and EGFR. The correlation between EGFR and AREG expression was statistically insignificant (Φ = 0.039, *P* = 0.709).

### Prognostic Factors Affecting DFS and OS

The median follow-up for DFS and OS was 9.50 months (range, 1–36 months) and 17.50 months (range, 2–48 months), respectively. The mean DFS and OS was 11.47 months and 17.71 months, respectively.

**Fig. 2:**
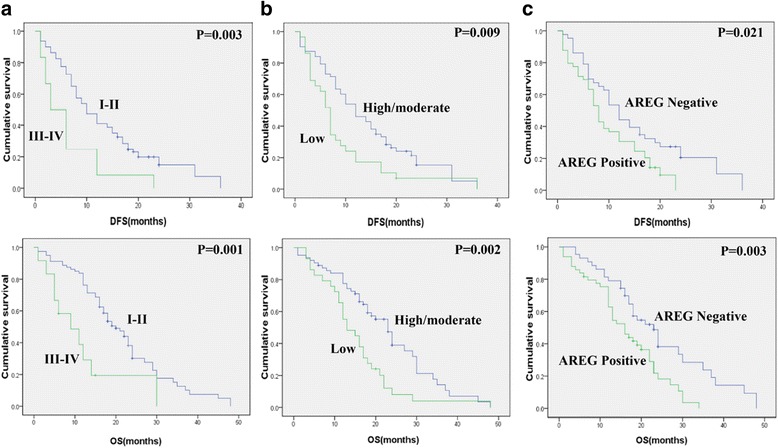
Predictors of DFS and OS in PDAC. Kaplan-Meier curves for DFS (*top*) and OS (*bottom*) of PDAC patients with TNM stage (**a**), Tumor differentiation (**b**) and AREG expression (**c**). P values for comparison of groups using the log-rank test are given

In the univariate survival analysis, high TNM stage (*P* = 0.003 and *P* = 0.001), poor tumor differentiation (*P* = 0.009 and *P* = 0.002) and AREG expression (*P* = 0.021 and *P* = 0.003) were significant adverse prognostic factors for DFS and OS, respectively (Table 3, Fig. 2). PDAC patients with positive expression for AREG showed significantly shorter DFS and OS (AREG-positive, 8 months vs. AREG-negative, 12 months; AREG-positive, 16 months vs. AREG-negative, 23 months). To analyze the prognostic significance of the AREG/EGFR interaction, the study population was divided into three groups: an AREG/EGFR co-expression group, a single-positive group and a dual-negative group. Using the log-rank test, however, none of these groups showed significant differences in DFS or OS (data not shown).

**Table 3:** Univariate analysis for disease-free survival and overall survival

Parameter	DFS			OS		
	HR	95 % CI	*P*	HR	95 % CI	*P*
Age(years)			0.944			0.84
≥ 60 (vs. < 60)	0.985	0.632–1.537		0.954	0.594–1.534	
Gender			0.456			0.416
Female (vs. Male)	1.176	0.754–1.835		1.21	0.754–1.943	
Tumor sites			0.69			0.629
Body/tail (vs. Head)	0.918	0.589–1.431		0.893	0.555–1.439	
Tumor size			0.181			0.052
T3-4 (vs. T1-2)	1.379	0.836–2.274		1.658	0.967–2.843	
TNM stage			0.003			0.001
III-IV (vs. I-II)	2.399	1.284–4.482		2.849	1.428–5.683	
Tumor differentiation			0.009			0.002
Poor (vs. Well/moderate)	1.784	1.118–2.846		2.059	1.269–3.34	
Resection margins			0.282			0.089
Positive (vs. Negative)	1.338	0.77–2.326		1.601	0.912–2.81	
Lymph node metastasis			0.314			0.06
Yes (vs. No)	1.238	0.797–1.923		1.535	0.961–2.453	
AREG expression			0.021			0.003
Positive (vs. Negative)	1.688	1.059–2.691		2.043	1.238–3.374	
EGFRvIII expression			0.466			0.466
Positive (vs. Negative)	1.256	0.663–2.381		1.292	0.637–2.623	
AREG/EGFR coexpression			0.08			0.166
Negative (vs. Positive)	0.853	0.646–1.128		0.831	0.619–1.115

The hazard ratio (HR) was estimated by Cox regression, AREG expression was found to increase the HR for recurrence (HR = 1.688, 1.059–2.691) and death (HR = 2.043, 1.238–3.374) (Table 3). Multivariate analysis was performed to find independent factors that could affect DFS and OS. Factors at the 0.10 level in the univariate analysis (TNM stage, tumor differentiation, tumor size, Resection margins, lymph node metastasis, AREG expression and AREG/EGFR co-expression) were entered into a multivariate survival analysis. Poor tumor differentiation was an independent unfavorable prognostic factor for DFS (HR = 1.785, *P* = 0.021) and OS (HR = 2.125, *P* = 0.004). Moreover, AREG expression (HR = 1.822, *P* = 0.03), high TNM stage (HR = 225, *P* = 0.03), and positive resection margins (HR = 1.84, *P* = 0.045) were all independent prognostic indicators for poor OS (Table 4).

**Table 4:** Multivariate analysis for disease-free survival and overall survival

Parameter	HR	95 % CI	*P*
Disease-free survival: Cox regression model			
TNM stage			0.06
III-IV (vs. I-II)	2.528	1.299–4.921	
Tumor differentiation			0.021
Poor (vs. Well/moderate)	1.785	1.089–2.925	
AREG expression			0.168
Positive (vs. Negative)	1.418	0.863–2.331	
AREG/EGFR coexpression			0.587
Negative (vs. Positive)	0.922	0.687–1.237	
Overall survival: Cox regression model			
Tumor size			0.485
T3-4 (vs. T1-2)	1.24	0.677–2.271	
TNM stage			0.03
III-IV (vs. I-II)	2.25	1.081–4.684	
Tumor differentiation			0.004
Poor (vs. Well/moderate)	2.125	1.277–3.537	
Resection margins			0.045
Positive (vs. Negative)	1.84	1.277–3.537	
Lymph node metastasis			0.417
Yes (vs. No)	1.233	0.743–2.048	
AREG expression			0.03
Positive (vs. Negative)	1.822	1.058–3.137	

## Discussion

Unfortunately, PDAC is associated with a largely unfavorable outcome and aggressive tumor biology. Tumor size, lymph node involvement and the status of the resection margin are traditional prognostic factors, although they are not adequate to distinguish between patients with a high and low risk of disease recurrence and metastasis. Our study is the first to simultaneously investigate the clinicopathological and prognostic significance of EGFRvIII (the most common mutated variant of EGFR) expression, AREG (EGFR ligand) expression and AREG/EGFR co-expression in PDAC. In our study, we showed that AREG/EGFR co-expression were associated with poor tumor differentiation. Also, AREG expression in PDAC was an independent prognostic indicator of poor OS according to our multivariate survival analysis.

According to previous immunohistochemical studies, EGFR is expressed in 23.9–68.4 % of PDAC samples [12, 15]. Handra et al. [16] found that tumor expression of EGFR was associated with clinical response but not outcome in PDAC. Funatomi et al. demonstrated the existence of an autoregulated AREG/EGFR feedback loop in pancreatic cancer [17]. After binding to EGFR, AREG stimulation of the intrinsic tyrosine kinase activity of EGFR induces a complex cascade of phosphorylation and activation events that determine cell proliferation, differentiation, and tumor development [18]. We found that the expression of AREG/EGFR co-expression were associated with poor tumor differentiation, which is consistent with previous studies.

In recent years, investigators have increasingly recognized the critical role of EGFRvIII in tumor carcinogenesis [19–21]. To the best of our knowledge, no published study has correlated the expression of EGFRvIII with prognosis in patients with pancreatic cancer. Evaluation of the expression of EGFRvIII in PDAC could provide additional knowledge concerning the complex mechanism of EGFR signaling in PDAC. EGFRvIII expression occurs at an overall frequency of 25–64 % when assessed by multiple techniques in GBM [20, 21]. Using immunohistochemistry, our study showed that 13 % (12 of 92) of PDAC patients were positive for EGFRvIII. Tinhofer et al. [10] found that expression of EGFRvIII was detected in 17 % of SCCHN patients, expression of EGFRvIII was significantly associated with shortened PFS but not with OS. In GBM, EGFRvIII expression was associated with poor prognosis [20, 21]. However, our study suggests that EGFRvIII expression has little prognostic significance on survival in PDAC patients. Thus, the prognostic significance of EGFRvIII expression in PDAC remains unclear and requires further clarification.

AREG has been recognized as an oncogenic factor for more than 20 years. The role of AREG in cancer development and progression is supported by clinical data showing that AREG can serve as a prognostic and/or a predictive biomarker [22–26]. Masago et al. [24] found that high serum levels of AREG and TGF-α were predictors of poor prognosis in patients with advanced non-squamous, non-small cell lung cancer (NSCLC). Similarly, AREG expression was shown to be independently associated with a reduced OS in a multivariate analysis of 195 patients with stages I-III NSCLC [25]. Moreover, the concomitant expression of AREG and EGFR was associated with enhanced tumor aggressiveness and shorter survival periods following tumor resection in PDAC [26]. The results from in vitro and in vivo studies have demonstrated that aberrantly activated AREG-EGFR signaling is required for CRTC1-MAML2-positive MEC (mucoepidermoid carcinoma) cell growth and survival. In particular, CRTC1-MAML2-positive MEC cells are highly sensitive to EGFR signaling inhibition, which suggests that EGFR-targeted therapies may benefit patients with MEC [27]. Consistent with these results, our study demonstrated that high AREG expression was an independent prognosticator of poor OS in PDAC. We also found that AREG/EGFR co-expression was associated with poor tumor differentiation, which is similar to that demonstrated in previous studies [22–26]. Recently, one study with conflicting results reported that the negative expression of AREG and positive expression of MMP-2 were hallmarks of tumor biology in PDAC patients [11]. Park et al. enrolled 88 PDAC patients and stained EGFR, AREG, VEGF, p-c-met, MMP2, MMP7, MMP9, CXCR3, and CXCR4 antibodies on tissue microarray (TMA) [11]. On the contrary, we stained our markers on whole tissue sections, which provided a wider scope and improved perception of tumor heterogeneity, eliminating potential bias. Compared with their uniform interpretation criteria, we adopted different criteria for positivity membranous staining and cytoplasmic staining. However, both studies were subjected to several limitations including limited sample size and lack the in-depth investigation into the pro-oncogenic mechanism of AREG in PDAC.

Numerous researchers have reported that AREG was correlated with invasion and distant metastases in multiple malignancies [28–30]. Increasing evidences revealed that the essential role of EMT in the local invasion and metastasis of pancreatic cancer. To study the role of AREG in the EMT of PDAC may provide insights into the tumor biology of PDAC cell migration and invasion for inspiration.

## Conclusions

Our results showed that although no significant association was observed between AREG expression alone and the clinicopathological characteristics in PDAC, there was a significant association between AREG/EGFR co-expression and poor tumor differentiation. Moreover, AREG expression, poor tumor differentiation, high TNM stage, and positive resection margins were all independent prognostic indicators for poor OS. Our data indicate that AREG expression identifies a subset of PDAC patients with more aggressive tumor characteristics and a significantly worse prognosis, indicating that AREG may serve as an attractive therapeutic strategy for PDAC.

## Abbreviations

EGFR, epidermal growth factor receptor; EGFRvIII, epidermal growth factor receptor variant III; AREG, amphiregulin; PDAC, pancreatic ductal adenocarcinoma; GBM, glioblastoma multiforme; SCCHN, squamous cell carcinoma of the head and neck; NSCLC, non-small cell lung cancer; CT, chemotherapy; RT, radiotherapy; DFS, disease-free survival; OS, overall survival

## Additional file

Additional file 1: Figure S1. AREG RNA and protein levels in pancreatic cancer cells and pancreatic stellate cells. A, AREG mRNA levels in pancreatic cancer cells and pancreatic stellate cells. B, AREG protein levels in pancreatic cancer cells and pancreatic stellate cells, * indicates a *P* < 0.05. (DOC 718 kb)
