# Supplementary material for: Expression of amphiregulin predicts poor outcome in patients with pancreatic ductal adenocarcinoma
Source: Diagn Pathol. 2016 Jul 8;11:60. doi: 10.1186/s13000-016-0512-4 (PMC4938900; doi:10.1186/s13000-016-0512-4)
Supplement: Additional file 1: Figure S1. — AREG RNA and protein levels in pancreatic cancer cells and pancreatic stellate cells. A, AREG mRNA levels in pancreatic cancer cells and pancreatic stellate cells. B, AREG protein levels in pancreatic cancer cells and pancreatic stellate cells, * indicates a P < 0.05. (DOC 718 kb) [file 13000_2016_512_MOESM1_ESM.doc]

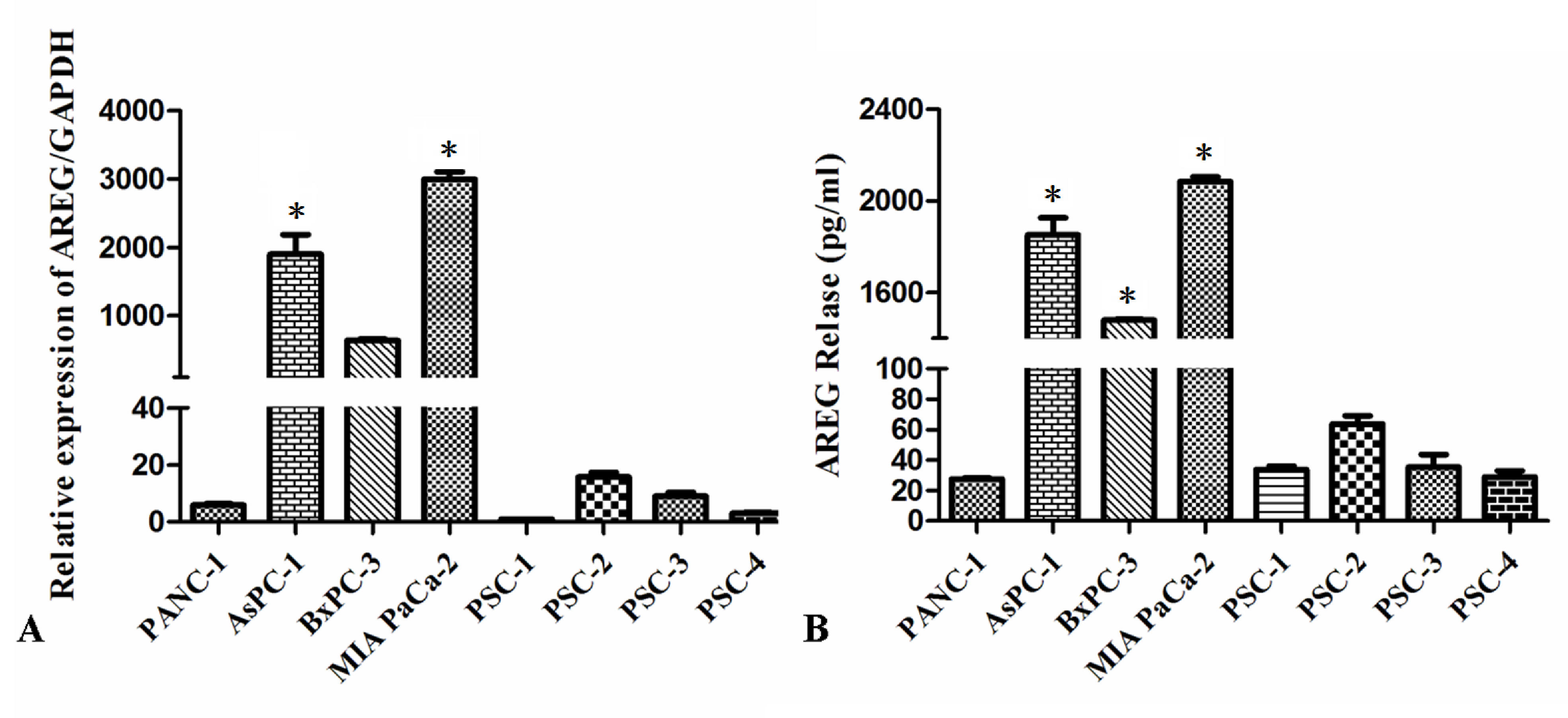


**Supplement Figure 1. AREG RNA and protein levels in pancreatic cancer cells and pancreatic stellate cells.** A, AREG mRNA levels in pancreatic cancer cells and pancreatic stellate cells. B, AREG protein levels in pancreatic cancer cells and pancreatic stellate cells, * indicates a P<0.05.
